# General Issues in Clinical Research of Acupuncture and In Vitro Fertilization

**DOI:** 10.1155/2020/3460641

**Published:** 2020-04-13

**Authors:** Cuihong Zheng, Xiaoyang Wan, Xiaohu Xu, Mingmin Zhang, Guangying Huang, Wei Wang

**Affiliations:** ^1^Institute of Integrated Traditional Chinese and Western Medicine, Tongji Hospital of Tongji Medical College, Huazhong University of Science and Technology, No. 1095 Jiefang Avenue, Wuhan 430030, China; ^2^Institute of Infectious Disease, Tongji Hospital of Tongji Medical College, Huazhong University of Science and Technology, No. 1095 Jiefang Avenue, Wuhan 430030, China; ^3^Department of Neurology, Tongji Hospital of Tongji Medical College, Huazhong University of Science and Technology, No. 1095 Jiefang Avenue, Wuhan 430030, China

## Abstract

In vitro fertilization-embryo transfer (IVF-ET), a well-developed technology, provides the last possibility or hope for infertile women. However, the live birth rate per IVF cycle is still not satisfactory. Acupuncture is a frequently used adjunctive therapy drawing wide attention on improvement of IVF. Although so many randomized controlled trials have been evaluating the effect of acupuncture on IVF in the past 20 years, the exact efficacy of acupuncture on IVF is still controversy mainly because of no consensus on placebo control and acupuncture scheme. This paper focused on the general issues in clinical research of acupuncture and IVF and gave some suggestions for future directions.

## 1. Introduction

Infertility is a growing problem with rapid economic development, lifestyle changes, and deterioration of environmental pollution. In vitro fertilization-embryo transfer (IVF-ET), a well-developed technology, provides the last possibility or hope for pregnancy. However, the live birth rate per IVF cycle is still not satisfactory in that many patients cannot succeed even after several ETs [1]. Meanwhile, the latent safety problems associated with using large doses of ovulation stimulants to obtain more eggs for IVF cannot be ignored [2]. Furthermore, IVF is an expensive procedure that repeated cycles place enormous economic pressure on the patients and their families. Therefore, it is necessary to maximize the efficiency of the IVF procedure.

Complementary and alternative medical (CAM) treatments may be good options for patients to increase the success rate of IVF. Among these CAM treatments, acupuncture is a frequently used adjunctive therapy drawing wide attention on improvement of IVF. Albeit so many randomized controlled trials (RCTs) have been evaluating acupuncture in IVF in the past 20 years, the exact efficacy on IVF is still controversy mainly because of no consensus on placebo control and acupuncture scheme. Some studies suggested a positive impact from adding acupuncture to IVF [3–7], but others did not [8, 9]. Contradictory results were also shown in lines of systematic reviews and meta-analyses adding the complexity of acupuncture on IVF [10]. Particularly in recent years, some clinical studies appeared on the Journal of the American Medical Association (JAMA) reported no difference of the effect in between the real acupuncture and sham acupuncture [11, 12], adding doubt whether acupuncture influences IVF pregnancy or not.

Given the different conclusions on the acupuncture in IVF, this paper will focus on the general issues in design of clinical acupuncture research, especially for subfertility or IVF, and gave some suggestions for future directions in acupuncture and IVF.

## 2. General Issues in Clinical Research of Acupuncture and IVF

### 2.1. Difficulty of Placebo Acupuncture

Double blind RCTs are considered as the gold standard of evidence-based medicine for pharmaceutical trials because they can determine causality between the intervention and outcome. However, it is very challenging to design an ideal placebo or sham control in the clinical trials of acupuncture. First, it is not feasible to blind acupuncturists. Acupuncturists need to know which group is the real acupuncture treatment and which group is the control treatment, so as to correctly perform the acupuncture operation. Second, the control treatment must be indistinguishable and worthy of full trust. In the meantime, the control cannot produce any treatment effect.

At present, placebo acupuncture mainly contains deep acupuncture at nondisease-related acupoints or nonacupoints, superficial acupuncture at specific acupoints, nondisease-related acupoints, or nonacupoints, and skin surface stimulation at acupoints or nonacupoints [13]. Which kind of placebo acupuncture is ideal? Actually, no current placebo acupuncture meets the standard requirements of placebo methods. There are several inappropriate points of view about acupuncture or meridians.

First, most clinical studies of acupuncture ignored the fact that the meridian system includes the part of skin and then mistook that superficial acupuncture or skin surface stimulation at acupoints has no therapeutic effect. Actually, superficial acupuncture is an integral part of acupuncture. The ancient book “Suwen” has a record of “Disease has a nature of floating or sinking, and acupuncture should be superficial or deep accordingly”. So, if the nature of the disease is superficial, superficial acupuncture might approach better effect compared to deep acupuncture.

In order to let the participants not realize the difference between real acupuncture and placebo acupuncture, some researchers used Streitberger, Takakura, or Park placebo needles, etc., which are new types of nested blunt needles [14]. Though the blunt needle does not penetrate into the skin, the patients can feel pricking sensation during simulated acupuncture. The needle moves inside the handle and appears to be shortened. So, this kind of control is seemly indistinguishable and worthy of full trust. However, the noninvasive but pricking placebo acupuncture used at acupoints may also have elicited physiological effects similar to those of acupressure because it stimulates the skin, the component of acupoint, which has been shown to be an effective treatment for various conditions [15, 16].

Second, most researchers think that the specific effect can be avoided by puncture at points away from the acupoints. In fact, in addition to the 362 meridian acupoints of the textbook, there are so many extra acupoints, such as more than 700 Dong's extra acupoints with exact names and main functions [17], and there may be many other acupoints on the human body that have not been explored yet. Also, each acupoint has a domain. If a nonacupoint is too close to an acupoint, maybe there is no significant difference between the two effects. Given that so many known and unknown acupoints exist on the body, it is not very easy to define a real nonacupoint.

From the above analysis, all the present methods of the acupuncture placebo control are not ideal. The concept and use of the traditional placebo method are not fully applicable to acupuncture, as same as that placebo control cannot be used in surgical operations. Because of the fact that the sham acupuncture may be not an inert placebo but rather an active treatment that may affect the pregnancy outcome, using sham acupuncture as the control may confuse rather than clarify the effects of acupuncture for IVF [18]. So, sham acupuncture may be not necessary, especially for clinical studies that observe objective results, although it has often been used as the control in RCTs with subjective, patient-reported outcomes such as pain, which can be largely affected by judgments and expectations [19].

### 2.2. Safety of Acupuncture

Some clinical trials showed that there was no difference of acupuncture effect between acupoints and nonacupoints, so some researchers consider that the effect of acupuncture is a placebo effect, without acupoint specificity, and acupuncture at any point may be the same [20]. In fact, this is not the case. In Dieterle's study [5], during IVF, acupuncture was applied for 30 minutes immediately after ET and again 3 days later. The placebo acupuncture treatment was originally designed not to influence fertility at Sidu, Xiaoluo, Fengshi, Zhongdu, and Yanglingquan. The clinical pregnancy rates for the acupuncture group and control group were 33.6% and 15.6%, respectively. However, according to the German IVF/ICSI register (2003), the average clinical pregnancy rates for this age are 24.6% for IVF and 22.6% for ICSI, respectively. So, it indicates that placebo acupuncture at such points may have an adverse effect on the pregnancy rate.

In Craig's study [8], the protocol was based on Paulus protocol with the addition of Cv6 before and KI3 after transfer. Acupuncture was performed before and after ET at an offsite location. The control group underwent ET without any other intervention. The results showed that the clinical pregnancy rate was significantly higher in the control group than in the acupuncture group (69.6% vs. 43.8%, respectively). This indicated that acupuncture performed at offsite on the day of ET might be detrimental to the success of transfer pregnancy.

Westergaard performed acupuncture immediately before and after ET in acupuncture 1 and 2 groups, and one 25-minute session was performed 2 days later in the acupuncture 2 group [4]. The result showed that acupuncture on the day of ET significantly improved the reproductive outcome of IVF/ICSI compared with no acupuncture. However, repeating acupuncture on ET day 2 provided no additional beneficial effect but even a greater early pregnancy loss. This indicates that acupuncture at some points might be unsuitable after ET.

In Smith's study, the number of miscarriages among women receiving acupuncture was numerically higher than among women receiving sham control (22.8% vs. 11.6%), although the difference was not statistically significant (*P*=0.054). Adverse events were significantly greater in the acupuncture group compared with the sham control for discomfort on the day of ET (10.3% vs. 4.9%; *P*=0.01) and bruising (5.0% vs. 1.3%; *P*=0.02) [11].

Together, acupuncture is not necessarily beneficial and even has the opposite clinical effect. Therefore, acupoints should be selected based on syndrome differentiation of traditional Chinese medicine (TCM), and the operation should be performed gently and carefully.

### 2.3. Lack of Treatment Based on Syndrome Differentiation

Almost all of the acupuncture-IVF RCTs used a fixed acupuncture protocol for all patients, which means the lack of Chinese medicine diagnosis. TCM emphasizes treatment based on syndrome differentiation, which includes a complex diagnosis system. Patients with the same Chinese medicine diagnosis (the same syndrome) may have different biomedical diagnoses and clinical presentations but be treated with the same treatment because of the same Chinese medicine syndrome. Conversely, patients with the same biomedical diagnosis may have different Chinese medicine diagnoses (different syndromes) and would therefore receive different treatments. This means that it is the Chinese medicine diagnosis that determines the treatment, not the biomedical diagnosis.

However, it is difficult for an RCT to accommodate treatment based on syndrome differentiation. The most commonly used acupoints or acupuncture protocols are selected usually appropriate for the average patient. These average treatments are highly likely not the most optimal because they are not designed specifically for any of the participants in the trial.

### 2.4. Insufficient Acupuncture Dosage

The first and most commonly used acupuncture protocol for IVF is called “Paulus protocol” [3]. Paulus protocol is performed just twice, with one before and another after ET. In this trial, subjects that received acupuncture had a significantly higher clinical pregnancy rate of 42.5% compared to the controls of 26.3% who did not receive acupuncture. Surprisingly, many subsequent trials have failed to repeat this result [21–23], including Paulus himself [24]. Part of the reason for inability to repeat this result is due to the fact that the later studies performed a possible active placebo control.

The other important point is related to the acupuncture dosage. Although it is possible for patients to get beneficial effects from one or two treatments, it is difficult for a chronic long-term disorder. The nature and duration of a condition is a very important determining factor for the dosages of treatments required. Acute disorders of short duration in younger patients are most likely to respond to small dosages of acupuncture. Conversely chronic disorders of long-term duration in older patients need larger dosage of acupuncture [25]. Infertility is often associated with either significant previous gynecological issues (e.g., endometriosis, polycystic ovarian syndrome, and premature ovarian insufficiency) and/or with patients whose age (often late 30 s/early 40 s) brings significant additional physiological challenges. Therefore, most of them need larger dosage of acupuncture. A clinically valid dosage of acupuncture usually includes four to ten (or more) acupuncture points given in each of six (or more) acupuncture treatments [26]. However, most of the dosages of acupuncture in these trials including the one published in JAMA [11] were too less to completely correct the infertility state caused by long-term insufficiencies or imbalances.

## 3. Future Directions

Infertile patients, such as those with irregular menstruation, follicles growing slow, and anovulation, excluding the blockage of fallopian tube, can seek acupuncture to overcome infertility before IVF. This is common in China. We can also design some RCTs to evaluate the effect of acupuncture on infertility. For future clinical trials about acupuncture in IVF, some aspects should be considered:

1. Unified IVF plan. What needs to be verified is whether acupuncture can improve the success rate of IVF. Therefore, the IVF scheme should be unified relatively, because different IVF schemes, such as different follicular induction schemes, have different pregnancy rates [27].
2. Optimization of acupuncture scheme. The acupuncture scheme should be optimized according to the condition of patients. Some women only seek acupuncture treatment during an IVF cycle, and some receive the treatment before, during, and between IVF cycles. If the short-term treatment was beneficial for the reproductive system, it would be logical that the longer-term treatment throughout the entire IVF cycle or adding treatment before and between IVF cycles would also be beneficial [25]. It is noticeable that in Kong's program, the combination of traditional Chinese acupuncture and electroacupuncture, at least 12 times before ET, achieved a 81.8% success (twice the US average for IVF alone) [7], and in Magarelli's program that a total of 11 treatments resulted in 51% clinical pregnancy rate (37% for the control group).
3. Primary endpoints and secondary endpoints. We should focus on not only the effect of acupuncture on the success rate of IVF (as primary endpoints) but also the quantities and qualities of eggs and embryos, the thickness of endometrium, and so on, as secondary endpoints.
4. Comfort acupuncture. We noted that some trials indicated the acupuncture group had lower LBR odds than the Streitberger control group [21–23]. It might be due to (1) the acupressure effect of the sham control stimulation, (2) real acupuncture is often accompanied by some degree of discomfort or pain, and a portion of patients is afraid of acupuncture, which may do harm. The shortcoming of real acupuncture can be avoided by noninvasive stimulation, such as transcutaneous electrical acupoints stimulation (TEAS), and patients may find it more acceptable. So, we speculated TEAS could induce the same or better therapeutic effects [10].
5. In recent years, several articles have reported the positive role of TEAS in IVF [28–30], confirming our initial assumption and indicating that we should focus on comfort acupuncture for infertility, not like paralytic diseases, and the stronger the stimulation, the better the effect [31]. The patient should feel relaxed or unstressed and be adapted to the technique and acupuncturist before the IVF cycle begins. If acupuncture is done on the day of ET, it should ideally be done in the IVF center or at least with minimal travel to the acupuncturist.
6. Since the control of placebo acupuncture is not mature at present, it is not suggested to set an acupuncture placebo control as usual. This issue relates not only to acupuncture needles but also to other treatment devices that involve physical contact with the patient, such as injections, transcutaneous electrical nerve stimulation, manual therapy, and surgical interventions. Placebo devices, including placebo injections and placebo acupuncture needles, exhibit stronger effects than do oral placebo pills [32]. Therefore, a blank control needs to be set, or a blank control can be added when designing a placebo acupuncture control, which is conducive to analyzing and reflecting the real efficacy of acupuncture. The blank control includes no treatment controls, waiting list control, and treatment as usual, which all exhibit both specific advantages and pitfalls.

Actually, to some extent, acupuncture effect has included physical and mental treatment since ancient times. That means acupuncture treatment is a process of integral adjustment of body and mind, not just a physical stimulation at acupoints. The patient's recognition, anticipation, attention, preference, and doctor-patient communication may be influencing factors in the production of the total acupuncture effect. Therefore, in acupuncture clinical practice, a good doctor pays attention to communication, emphasizes the physical and mental treatment, and not deliberately excludes all consolation effect. That might be part of the reason why the effect of TCM including acupuncture in practice is relatively satisfactory, which directly leads to the popularity of TCM in the reproductive area.

## References

[1] Smith C. A., De Lacey S., Chapman M. (2019). The effects of acupuncture on the secondary outcomes of anxiety and quality of life for women undergoing IVF: a randomized controlled trial. *Acta Obstetricia et Gynecologica Scandinavica*.

[2] Engmann L., DiLuigi A., Schmidt D., Nulsen J., Maier D., Benadiva C. (2008). The use of gonadotropin-releasing hormone (GnRH) agonist to induce oocyte maturation after cotreatment with GnRH antagonist in high-risk patients undergoing in vitro fertilization prevents the risk of ovarian hyperstimulation syndrome: a prospective randomized controlled study. *Fertility and Sterility*.

[3] Paulus W. E., Zhang M., Strehler E., El-Danasouri I., Sterzik K. (2002). Influence of acupuncture on the pregnancy rate in patients who undergo assisted reproduction therapy. *Fertility and Sterility*.

[4] Westergaard L. G., Mao Q., Krogslund M., Sandrini S., Lenz S., Grinsted J. (2006). Acupuncture on the day of embryo transfer significantly improves the reproductive outcome in infertile women: a prospective, randomized trial. *Fertility and Sterility*.

[5] Dieterle S., Ying G., Hatzmann W., Neuer A. (2006). Effect of acupuncture on the outcome of in vitro fertilization and intracytoplasmic sperm injection: a randomized, prospective, controlled clinical study. *Fertility and Sterility*.

[6] Magarelli P. C., Cridennda D. K., Cohen M. (2009). Changes in serum cortisol and prolactin associated with acupuncture during controlled ovarian hyperstimulation in women undergoing in vitro fertilization-embryo transfer treatment. *Fertility and Sterility*.

[7] Kong S., Hughes A. (2009). Acupuncture as an adjunct to in vitro fertilization: a randomized trial. *Medical Acupuncture*.

[8] Craig L. B., Criniti A. R., Hansen K. R., Marshall L. A., Soules M. R. (2007). Acupuncture lowers pregnancy rates when performed before and after embryo transfer. *Fertility and Sterility*.

[9] Domar A. D., Meshay I., Kelliher J., Alper M., Powers R. D. (2009). The impact of acupuncture on in vitro fertilization outcome. *Fertility and Sterility*.

[10] Zheng C. H., Huang G. Y., Zhang M. M., Wang W. (2012). Effects of acupuncture on pregnancy rates in women undergoing in vitro fertilization: a systematic review and meta-analysis. *Fertility and Sterility*.

[11] Smith C. A., De Lacey S., Chapman M. (2018). Effect of acupuncture vs. sham acupuncture on live births among women undergoing in vitro fertilization. *Journal of the American Medical Association*.

[12] Wu X.-K., Stener-Victorin E., Zhang H. (2017). Acupuncture for infertility in polycystic ovary syndrome-reply. *Journal of the American Medical Association*.

[13] Wu J.-N., Qin Z.-S., Liu Z.-S. (2017). Pivotal factors concerned in design of acupuncture clinical research: from two articles in JAMA. *Chinese Journal of Integrative Medicine*.

[14] Zhang C. S., Tan H. Y., Zhang G. S., Zhang A. L., Xue C. C., Xie Y. M. (2015). Placebo devices as effective control methods in acupuncture clinical trials: a systematic review. *PLoS One*.

[15] Hjelmstedt A., Shenoy S. T., Stener-Victorin E. (2010). Acupressure to reduce labor pain: a randomized controlled trial. *Acta Obstetricia et Gynecologica Scandinavica*.

[16] Hsieh L. L.-C., Kuo C.-H., Lee L. H., Yen A. M.-F., Chien K.-L., Chen T. H.-H. (2006). Treatment of low back pain by acupressure and physical therapy: randomised controlled trial. *British Medical Journal*.

[17] Zuo C. (2013). Chinese Dong‘s extra acupoints school. *World Journal of Acupuncture-Moxibustion*.

[18] Manheimer E. (2011). Selecting a control for in vitro fertilization and acupuncture randomized controlled trials (RCTs): how sham controls may unnecessarily complicate the RCT evidence base. *Fertility and Sterility*.

[19] Flum D. R. (2006). Interpreting surgical trials with subjective outcomes. *Journal of the American Medical Association*.

[20] Langevin H. M., Hammerschlag R., Lao L., Napadow V., Schnyer R. N., Sherman K. J. (2006). Controversies in acupuncture research: selection of controls and outcome measures in acupuncture clinical trials. *The Journal of Alternative and Complementary Medicine*.

[21] Smith C., Coyle M., Norman R. J. (2006). Influence of acupuncture stimulation on pregnancy rates for women undergoing embryo transfer. *Fertility and Sterility*.

[22] Andersen D., Løssl K., Nyboe Andersen A. (2010). Acupuncture on the day of embryo transfer: a randomized controlled trial of 635 patients. *Reproductive BioMedicine Online*.

[23] So E. W. S., Ng E. H. Y., Wong Y. Y., Yeung W. S. B., Ho P. C. (2010). Acupuncture for frozen-thawed embryo transfer cycles: a double-blind randomized controlled trial. *Reproductive BioMedicine Online*.

[24] Paulus W., Zhang M., Sterzik K., Seybold B. (2003). Placebo-controlled trail of acupuncture effects in assisted reproduction therapy. *O-052 Abstracts of the 19th Annual Meeting of the ESHRE*.

[25] Anderson B., Rosenthal L. (2013). Acupuncture and in vitro fertilization: critique of the evidence and application to clinical practice. *Complementary Therapies in Clinical Practice*.

[26] Anderson B., Nielsen A., McKee D., Jeffres A., Kligler B. (2012). Acupuncture and heart rate variability: a systems level approach to understanding mechanism. *Explore*.

[27] Xu Y., Zhang Y. S., Zhu D. Y., Zhai X. H., Wu F. X., Wang A. C. (2018). Influence of GnRH antagonist in reproductive women on in vitro fertilization and embryo transfer in fresh cycles. *Biomedical Reports*.

[28] Zheng Y., Feng X., Mi H. (2015). Effects of transcutaneous electrical acupoint stimulation on ovarian reserve of patients with diminished ovarian reserve inin vitrofertilization and embryo transfer cycles. *Journal of Obstetrics and Gynaecology Research*.

[29] Shuai Z., Lian F., Li P., Yang W. (2015). Effect of transcutaneous electrical acupuncture point stimulation on endometrial receptivity in women undergoing frozen-thawed embryo transfer: a single-blind prospective randomised controlled trial. *Acupuncture in Medicine*.

[30] Shuai Z., Li X., Tang X., Lian F., Sun Z. (2019). Transcutaneous electrical acupuncture point stimulation improves pregnancy outcomes in patients with recurrent implantation failure undergoing in vitro fertilisation and embryo transfer: a prospective, randomised trial. *Acupuncture in Medicine*.

[31] Xu S.-B., Huang B., Zhang C.-Y. (2013). Effectiveness of strengthened stimulation during acupuncture for the treatment of Bell palsy: a randomized controlled trial. *Canadian Medical Association Journal*.

[32] Hróbjartsson A., Gøtzsche P. C. (2010). Placebo interventions for all clinical conditions. *Cochrane Database of Systematic Reviews*.

